# Mathematical modeling of multi-drugs therapy: a challenge for determining the optimal combinations of antiviral drugs

**DOI:** 10.1186/1742-4682-11-41

**Published:** 2014-09-25

**Authors:** Yoshiki Koizumi, Shingo Iwami

**Affiliations:** School of Medicine, College of Medical, Pharmaceutical and Health Sciences, Kanazawa University, 13-1 Takaramachi, Kanazawa-shi, Ishikawa, 920-8640 Japan; Department of Biology, Faculty of Sciences, Kyushu University, 6-10-1 Hakozaki, Higashi-ku, Fukuoka, 812-8581 Japan

**Keywords:** Antiviral therapy, Mathematical modeling, Drug combination theory, Virus dynamics

## Abstract

In the current era of antiviral drug therapy, combining multiple drugs is a primary approach for improving antiviral effects, reducing the doses of individual drugs, relieving the side effects of strong antiviral drugs, and preventing the emergence of drug-resistant viruses. Although a variety of new drugs have been developed for HIV, HCV and influenza virus, the optimal combinations of multiple drugs are incompletely understood. To optimize the benefits of multi-drugs combinations, we must investigate the interactions between the combined drugs and their target viruses. Mathematical models of viral infection dynamics provide an ideal tool for this purpose. Additionally, whether drug combinations computed by these models are synergistic can be assessed by two prominent drug combination theories, Loewe additivity and Bliss independence. By combining the mathematical modeling of virus dynamics with drug combination theories, we could show the principles by which drug combinations yield a synergistic effect. Here, we describe the theoretical aspects of multi-drugs therapy and discuss their application to antiviral research.

## Background

Several landmark mathematical modeling studies of anti-retroviral therapy were reported in 1995 [1–4]. Thereafter, mathematical modeling has provided quantitative insights into antiviral drugs targeting HIV [5–13], HCV [14–18], HBV [19, 20] and influenza virus [21, 22]. These studies have elucidated the key steps of viral replication cycles and viral infection dynamics, such as the half-life of viruses and how viruses replicate in cells (reviewed in [23–27]). Although mathematical viral models could also search for drug combinations that improve antiviral effects and prevent the emergence of drug-resistant viruses, this potential of viral modeling has yet to be properly explored. Multi-drug administration is a standard treatment for HIV and HCV infection, and is also required in HBV and influenza. The established combination drug therapy for HIV, known as highly active anti-retroviral therapy (HAART), remains prohibitively expensive in developing countries. Additionally, HIV patients receiving long-term HAART may experience severe side effects such as lipodystrophy, hepatotoxicity, renal dysfunction, peripheral neuropathy and cardiovascular diseases (reviewed in [28]). By optimizing drug combinations, we could reduce the doses of individual drugs, thereby lowering the cost of treatment, reducing side effects, enhancing the antiviral effects and reducing the risk of drug-resistant viruses.

To accelerate the above benefits of drug combinations, we need to know whether or not a drug combination exerts a synergistic effect (reviewed in [29, 30]). A non-synergistic drug combination requires a larger than expected dose to achieve a regular antiviral effect. Although multi-drug therapy is standard practice, the complexity of the combined drugs’ responses is improperly understood, and rendered counter-intuitive by drug absorption, distribution, metabolism, and excretion through drug transporters [28]. To overcome these difficulties, comprehensive trials (or experiments) of various combinations and doses of drugs have been required. The combined effect has been investigated by empirical methods such as Loewe additivity [29, 31] and Bliss independence [29, 32]. Both theories have evolved from pharmacological research, and are used to classify combination effects as synergistic, additive or antagonistic [29, 33, 34]. These empirical frameworks have become widely accepted in pharmacology and have provided quantitative insight into drug combinations. However, because their underlying mechanics are unknown, the frameworks cannot provide reasons for a given combination effect (e.g. why is this drug combination additive, whereas that combination is antagonistic?). Showing the mechanisms of drug combination effects is expected to remarkably advance the development of multicomponent therapeutics. Thus, in addition to judging drug combination effects, knowledge of the mechanism is crucial to fundamental antiviral research.

Here we discuss two primary concepts of drug combination theory: Loewe additivity and Bliss independence. We apply these models to viral replication in a host cell, and discuss their utility for understanding two-drug interactions and viral dynamics. Finally, we discuss how drug combination theory might be applied to cancer chemotherapies and antiviral therapies. Combining mathematical models of viral replication with drug combination theory, we can establish an efficient framework for optimizing drug combinations.

### Basic drug combination theory: Loewe additivity

Loewe additivity is regarded as a primary criterion for evaluating drug combination effects [29]. To conceptualize Loewe additivity, let us consider the simplest situation, in which a combination of drugs A and B has a synergistic, additive or antagonistic effect. If the effect is additive, the Loewe additivity is defined as [31]:
1

where  and  are the concentrations of drugs A and B respectively in the combined dose, and *C*_*A*_ and *C*_*B*_ are the respective concentrations of drugs A and B that produce the same effect as the drug combination. That is, the Loewe additivity specifies the concentration ratio of a single drug and its combination with another drug. Note that Loewe additivity assumes that two drugs target the same molecule or pathway. If two drugs do not mutually interact, they can be related through the combination index (*CI*) based on the mass action law derived by Chou and Talalay [35]:
2

where the right hand side of Eq. (2) is identical to the left side of Eq. (1). When CI < 1, the relationship is synergetic, when CI = 1, it is additive, and when CI > 1, it is antagonistic. For example, suppose that 4 μM of drug A and 5 μM of drug B exert the same effect as a combination of 1 μM of A and 2 μM of B. Substituting these concentrations into Eq. (2), we obtain *CI* = 0.65 (=1/4 + 2/5 < 1), implying that the drug combination is synergistic. Note that the *CI* does not express the extent of synergy or antagonism; it is merely a criterion that separates the two behaviors without quantifying them.

To evaluate a drug combination effect by the *CI*, we require dose–response curves of drugs A and B, from which we can determine  and . The effect of a single drug *E* is modeled by the Hill function as follows:
3

where *E*_*max*_ is the maximum effect, *c* is the drug concentration, *h* is the Hill coefficient that determines the steepness of the dose–response curve, and *IC*_50_ is the concentration at which *E* exerts 50% of its maximum effect. Substituting Eq. (3) into Eq. (1) and rearranging in the case of *E*_*max*_ = 1, we obtain:
4

Numerically solving Eq. (4) for *E*, we can predict the additive effect at any drug concentrations  and [35, 36]. The following section explains an example of applying Loewe additivity to estimate rational drug combinations and optimal doses.

### Application of Loewe additivity for estimating rational drug combinations and optimal doses

**Figure 1:**
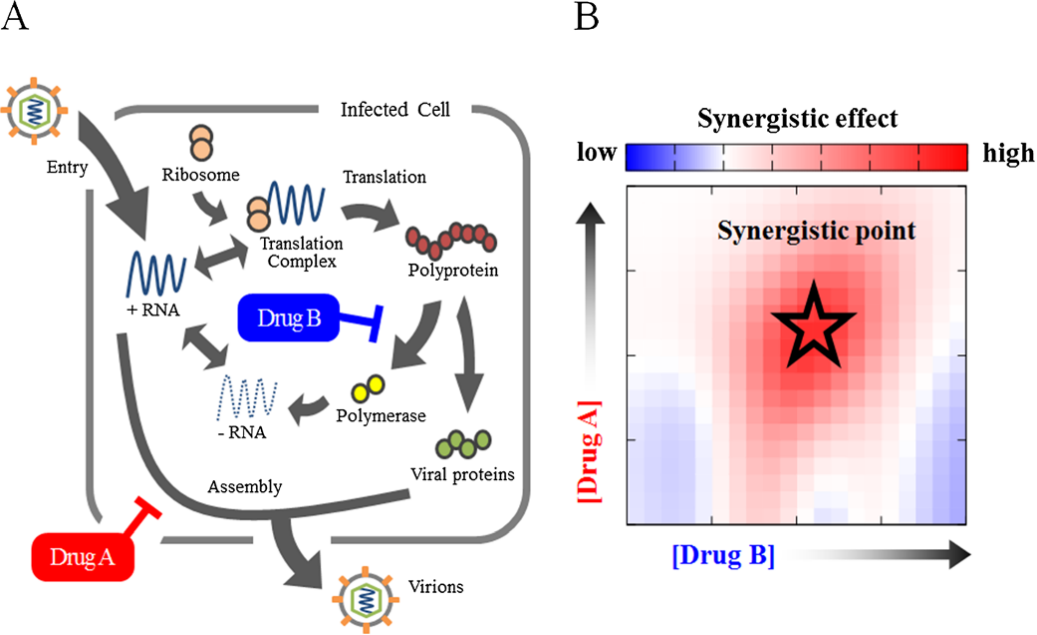
**Estimating the optimal drug combination from the mechanism of drug effects on viral replication. A)** Conceptual diagram showing the effects of two anti-HCV drug effects on HCV replication. Drug A inhibits virus release, and drug B inhibits the production of viral polymerase. To calculate the effect of co-administered A and B on HCV production, the dynamics of each viral component during the replication are expressed by differential equations [37–40]. The presence of both drugs decreases the parameters, a production rate of viral proteins and a releasing rate of virions. **B)** Estimation of the optimal dose point in a drug combination. Each point (corresponding to a specific dose) denotes the difference between the effects of the combined drugs obtained by modeling the viral dynamics and the additive effects calculated by Loewe additivity. A positive (red) difference indicates that the drug combination surpasses the expected additive effect. At such synergistic dose points, the antiviral effect can be achieved by administering the drug in combination at doses below the single drug dose (the synergistic point).

We here explain the usefulness of combining the Loewe additivity with mathematical modeling of hepatitis C virus (HCV) replication. The aim is to optimize the antiviral drug combination. Combining multiple drugs such as interferon-α and ribavirin is a standard therapy for enhancing the antiviral effects and reducing the risk of drug-resistant viruses. However, the conventional anti-HCV drugs fail to eradicate HCV genotype 1 in about 40% patients and may cause severe side effects. To overcome these problems, researchers have developed and administered direct acting antiviral (DAA) drugs that target viral-specific proteins such as viral proteases, as adjuncts to conventional anti-HCV drugs. To understand the principle behind optimal drug combinations and doses, we focused on the essential features of HCV replication and the mechanisms of the antiviral drugs (Figure 1A). Using previous mathematical models of HCV replication in a host cell [37–40], a modified mathematical model accounting for the mechanisms of drug actions would quantitatively simulate the anti-HCV effects at any dose of a drug combination. The effects of the drug combination at specific doses could then be evaluated by the Loewe additivity. In this way, we could estimate the synergistic dose point of a drug combination that enhances the anti-HCV effects (Figure 1B). The above analysis could predict the optimal drug combination that both reduces the total dose of each drug and enhances their anti-HCV effects.

### The alternative drug combination theory: Bliss independence

Bliss independence is the other major criterion for clarifying drug combination effects [32]. Bliss independence defines the expectation of a combined drug effect, calculated by multiplying the probabilities of the individual drugs. It assumes that each drug independently targets a different stage of viral replication via a different mechanism, with no interaction between the drug actions. Bliss independence expresses the unaffected probability of a two-drug combination *U*_*AB*_ as the product of the unaffected probabilities *U*_*A*_ and *U*_*B*_ of drugs A and B:
5

Intuitively, Eq. (5) implies that targets bypassed by drug A will be intercepted by drug B. The total probability of missed targets is obtained by multiplying the probabilities of the unaffected targets. Substituting *U* by 1 - *P*, where *P* denotes the probability of an intercepted target, Eq. (5) can be expressed as (1 - *P*_*AB*_) = (1 - *P*_*A*_) × (1 - *P*_*B*_), or
6

If the combined effect of the single drug doses is consistent with Eq. (6), this drug combination is evaluated as additive according to the Bliss independence measure. For instance, suppose that drugs A and B exhibit 50% and 60% inhibition, respectively. The Bliss independence predicts that drugs A and B will exert a combined inhibitory effect of 80%, calculated as *P*_*AB*_ = 0.5 + 0.6 - 0.5 × 0.6 = 0.8.

Unlike the Loewe additivity, the Bliss independence can evaluate the effect of a drug combination without requiring dose–response curves of the single drugs. If we attempt to assess a drug combination by the Loewe additivity, we must measure the effects at several single doses for constructing the dose–response curve. In contrast, the Bliss independence can assess the effects of a drug combination from a single point. Thus, the Bliss independence is useful if experimental data are limited. Additionally, in specific cases, the Bliss independence and Loewe additivity can be rendered equivalent using the Hill function Eq. (3) [41]. The following section explains an example of applying Bliss independence to quantifying the antiviral effects of intrinsic factors on HIV replication.

### Quantifying the antiviral effects of APOBEC3G on HIV replication by Bliss independence

**Figure 2:**
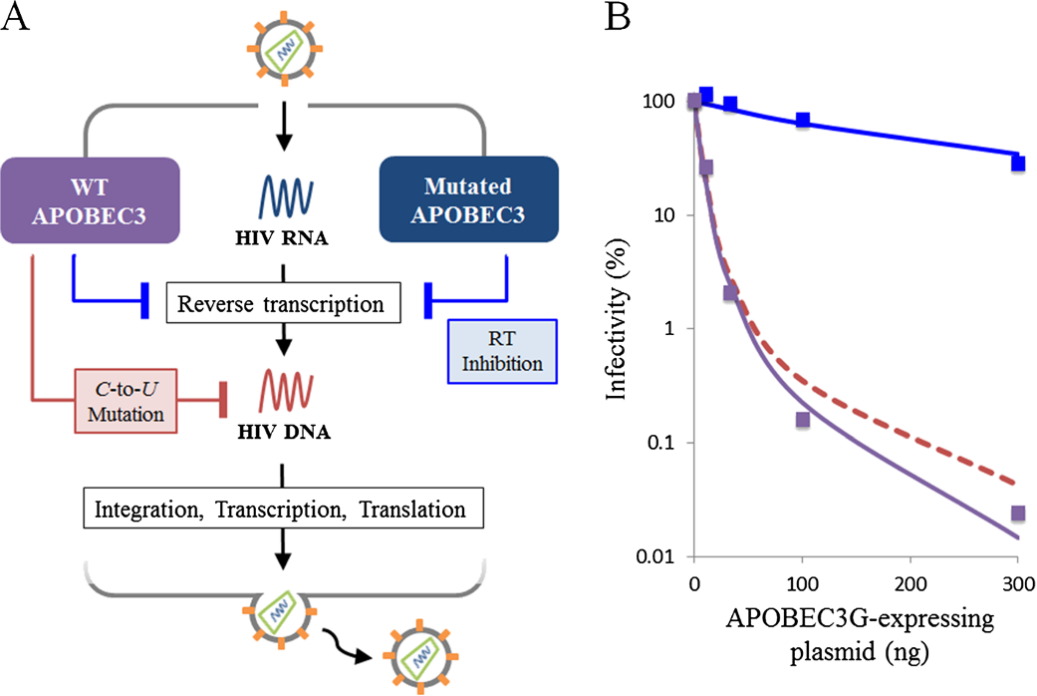
**Estimating the antiviral effects of C-to-U mutation by APOBEC3G on HIV production. A)** Mechanism of APOBEC3G inhibition in HIV replication. **B)** Quantification of antiviral effects of mutant APOBEC3G on HIV infection. HIV production reduces with increasing expression of APOBEC3G (dots: experimental data, solid lines: theoretical predictions, purple: WT APOBEC3G, blue: mutated APOBEC3G). Red dotted line shows the expected anti-HIV effect of C-to-U mutation activity, determined by Bliss independence.



The Bliss independence measure can predict antiviral effects as well as evaluate drug combinations. To illustrate this idea, we estimate the anti-HIV effect of a well-known antiviral host factor by Bliss independence [42]. Apolipoprotein B mRNA editing enzyme catalytic polypeptide-like 3G (APOBEC3G) suppresses HIV replication in cells by two main mechanisms: (1) inhibiting viral reverse transcriptase and (2) mutating cytidine (C) to uracil (U) in viral DNA by cytidine deaminase activity (Figure 2A). Although the anti-HIV effect of reverse transcriptase inhibition can be measured using a mutated APOBEC3G (with its C-to-U activity removed) (Figure 2A), the extent to which the mutation itself inhibits HIV replication cannot be experimentally determined. To quantify the anti-HIV effect of C-to-U mutation by APOBEC 3G, we can use Bliss independence to model the unaffected fractions of viral infectivity of wild-type (WT) and mutant APOBEC3G, denoted *f*_*WT*_ and *f*_*CI*_ respectively, in terms of the unaffected fractions of viral infectivity by reverse transcription inhibition and C-to-U mutation, *f*_*RT*_ and *f*_*Mu*_, as follows:

and


where *x* is the expression level of APOBEC3G, *h*_*RT*_ and *h*_*Mu*_ are Hill coefficients, and  and  are the expression levels required to achieve 50% inhibition by reverse transcription inhibition and C-to-U mutation, respectively. We estimated 4 parameters (*h*_*RT*_, *h*_*Mu*_, , and ) by fitting to experimental data of WT and mutated APOBEC3G (Figure 2B). Thus, the anti-HIV effect of C-to-U mutation by APOBEC3G can be quantitatively estimated by a Bliss independence-based modeling approach.

### Potential applications of drug combination theory

The Loewe additivity and Bliss independence form the basis of sophisticated protocols for assessing drug combinations, especially in cancer-targeted chemotherapy [33, 34]. Research on biological network systems, as well as the biochemical characteristics of drugs, has also unraveled the mechanisms by which drug combinations produce synergistic effects (reviewed in [41, 43–45]). Such research is important because (1) it facilitates rational drug discovery and (2) it predicts unknown connectivities in biological networks. Regarding the first point, the effects of a developing drug combined with conventional drugs are maximized by investigating biological networks, and rational drug targets are decided. For example, using computational modeling of cancer signaling networks and animal experiments, Kirouac *et al.*[46] identified the optimal inhibitor combination for suppressing the growth of *ERBB2*-amplified breast cancer. They adopted Bliss independence as a synergy criterion. Supplementing this computational approach with experimental data is useful for developing theoretically effective novel drugs. Regarding point (2), the biological networks of signaling pathways constructed by various components can be predicted from the dose–response shapes of drug combinations (reviewed in [47]). Lehar *et al.*[48] used several drug combination criteria, including Loewe additivity and Bliss independence, to connect biological components from the results of cellular responses to chemical combinations. This approach assumes that the dose–response shape of a drug combination depends on how drug targets are connected in a biological system.

**Figure 3:**
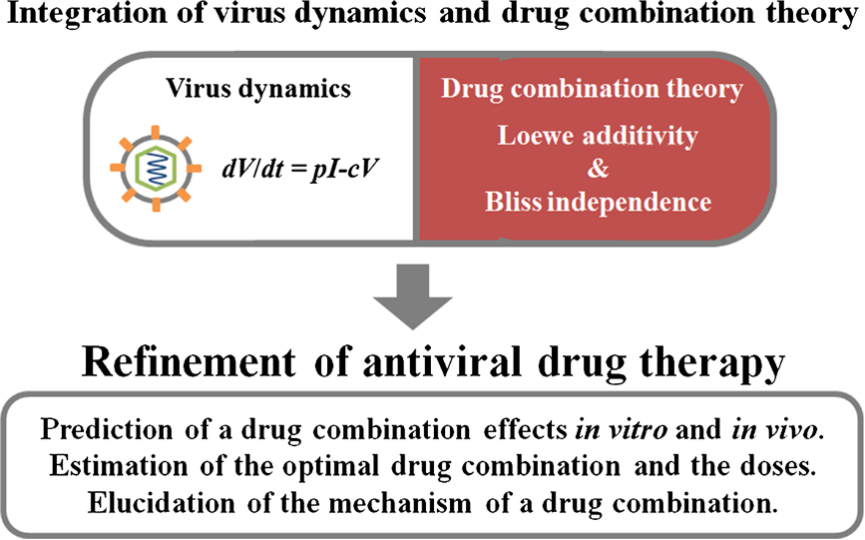
**Concept of integrating drug combination theory and viral dynamics.** The effect of a drug combination calculated by mathematical modeling of viral dynamics could be assessed by the Loewe additivity and Bliss independence. This approach could improve antiviral therapy by predicting the effect of a drug combination *in vivo* and *in vitro*, estimating the optimal drug dosage and combination, and discovering how the drugs interact.

The above research potentially benefits not only cancer chemotherapy, but also antiviral therapy development. For instance, Owens *et al.*[49] showed how HCV replication responds to chemical combinations that inhibit some enzymes involved in the sterol biosynthesis pathway. Supplementing computational simulation with *in vitro* experiments, they identified a rational inhibitor combination as a novel drug candidate. Mathematical models of HCV dynamics during drug therapy have also been investigated at the intracellular level [18], the intercellular level [14–17, 50, 51], and on a scale encompassing both levels [51–54]. These studies have quantitatively elucidated the properties of anti-HCV drugs such as interferon and protease inhibitors. Furthermore, by inputting clinical data to a mathematical model of HCV dynamics, Rong *et al.*[55] derived a mechanism by which drug-resistant viruses can rapidly emerge during single-drug therapy. Applying drug combination theories to these studies, mathematical models of HCV dynamics could be used to optimize the anti-HCV drug dosage and combination, thereby enhancing the anti-viral effect and reducing the risk of drug-resistant viruses (Figure 3).

## Conclusion

We have reviewed two major drug combination theories, Loewe additivity and Bliss independence, and discussed how combining these theories with mathematical modeling of viral dynamics might assist antiviral drug therapy. In addition, we have proposed an efficient framework for optimizing drug combinations and quantifying the anti-viral effect. Based on previous studies of computational virology, the integration of drug combination theory and dynamic modeling is a new approach with great potential for showing viral responses to drug combinations, and accelerating novel antiviral drug discovery.
